# To VAE or not to VAE: outcomes of radial scars/complex sclerosing lesions and papillary lesions without atypia in the King's College Hospital breast service (2017–2023)

**DOI:** 10.1111/his.70017

**Published:** 2025-10-02

**Authors:** Amy Llewellyn, Adam Brown, Juliet Morel, Kalnisha Naidoo

**Affiliations:** ^1^ Translational Pathology, Comprehensive Cancer Centre, School of Cancer and Pharmaceutical Sciences King's College London London UK; ^2^ Cellular Pathology King's College Hospital London UK; ^3^ Breast Radiology and Breast Screening Unit King's College Hospital London UK

**Keywords:** atypia, B3 lesions, papillary lesions, RS/CSL, uncertain malignant potential, upgrade risk

## Abstract

**Aims:**

Since there is currently limited data regarding the risk of malignancy that radial scars/complex sclerosing lesions (RS/CSL) or papillary lesions without atypia carry, we reviewed all such cases treated at King's College Hospital between January 2017 and June 2023 to determine the upgrade rates following immediate excision and longer follow‐up.

**Methods and results:**

Patients were identified using electronic database searches. An ‘upgrade’ was defined as the presence of ductal carcinoma in situ (DCIS) or invasive breast carcinoma (IBC) on excision at the biopsy site. One hundred and two patients had RS/CSL (85% screen‐detected; 15% symptomatic). Only one (1%) of the 90 patients who underwent excision was upgraded to DCIS; none to IBC. On longer follow‐up, four patients (4%) developed ipsilateral DCIS/IBC, while one patient developed contralateral DCIS with microinvasion. Two hundred and twenty‐six patients had papillary lesions without atypia (42% screen‐detected; 58% symptomatic). Eight (4%) of the 179 patients who underwent excision were upgraded to DCIS; none to IBC. On longer follow‐up, one patient developed ipsilateral DCIS; another patient developed contralateral IBC. For both lesions, radiological size was not significantly associated with atypia/upgrade (*P* > 0.05; Mann–Whitney *U*‐test).

**Conclusion:**

Since RS/CSL without atypia carry a low upgrade risk (1%), these patients could avoid excision and be followed up with mammographic surveillance. However, further data are needed for this change in practice to be considered. Papillary lesions without atypia appear to be more heterogeneous in behaviour, carrying an upgrade risk of 4%. Current treatment guidelines should not change until we better understand the biology of these lesions.

## Graphical abstract

Radial scars/complex sclerosing lesions (RS/CSL) without atypia have a low upgrade rate (1%) and could possibly be monitored radiologically, thus avoiding excision. Papillary lesions without atypia have a higher upgrade rate (4%) and should continue to undergo vacuum excision until we better understand these heterogeneous lesions.

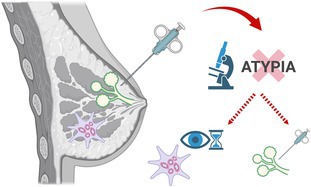

**Abbreviations**
CNB: core needle biopsy
CSL: complex sclerosing lesion
DCIS: ductal carcinoma in situ
KCH: King's College Hospital
MDT: multidisciplinary team
NHS: National Health Service
RS: radial scar
UK: United Kingdom
VAB: vacuum‐assisted biopsy
VAE: vacuum‐assisted excision

## Introduction

The optimal management of breast lesions of uncertain malignant potential (‘B3’) is complex and contentious. In the United Kingdom (UK), all breast core biopsies are classified histologically as ‘B1’ (normal) through ‘B5’ (malignant), and approximately 7% of breast biopsies are designated ‘B3’ (reviewed in^1^). This group includes heterogeneous lesions, like radial scars/complex sclerosing lesions (RS/CSL) and papillary lesions, that are often found adjacent to more sinister pathologies, but whose natural history in isolation is less well characterised.^1^, ^2^ Management guidelines vary according to the risk of upgrade to malignancy (i.e. ductal carcinoma in situ (DCIS) or invasive carcinoma) that each lesion carries on subsequent excision (surgical or vacuum‐assisted [VAE]).^1^ In particular, the presence or absence of epithelial atypia in the biopsy, which is routinely commented on by the reporting histopathologist, is central to guiding management decisions since atypical B3 lesions carry a significantly higher upgrade risk.^1^ How best to manage these lesions in the absence of atypia, however, is less clear, and as a result, excision might not be warranted in some patients at present.

Histologically, RS (≤10 mm in size) or CSL (>10 mm in size) comprise a central fibro‐elastotic core with radiating compressed, entrapped glandular structures. The entrapped ducts/lobules may show cystic changes, hyperplasia, and/or sclerosing adenosis.^3^ Radiologically, RS/CSL can mimic invasive carcinomas, as both present as stellate lesions causing architectural distortion. Reported upgrade rates for biopsies containing RS/CSL with atypia range from 25% to 36%,^1^, ^4^ justifying the current recommendation for subsequent excision.^1^ In contrast, reported upgrade rates for RS/CSL without atypia are low (<10%).^1^ Historically in the UK, these lesions were investigated by core needle biopsy (CNB; two or three 14‐gauge tissue cores), but since the publication of the NHS Breast Screening multidisciplinary working group guidelines in 2018, radiologists have also been using x‐ray guided, vacuum‐assisted biopsies (VAB; at least twelve 9 gauge tissue cores) to diagnose a subset of these lesions. There is some evidence to suggest that the VAB technique, by obtaining larger amounts of tissue for histological examination, decreases the upgrade risk in RS/CSL without atypia.^5^, ^6^

In papillary lesions, the risk of upgrade to malignancy also rises if atypia is present.^1^ At the benign end of the spectrum, intraductal papillomas arborise during growth, histologically comprising fibrovascular fronds with myoepithelial cells lining both the fronds within the lesion and the peripheral duct space. Clonality, visualised morphologically as monotonous cells initially with ER‐positive, low‐grade cytonuclear atypia can grow within these lesions, and once this atypia measures 3 mm, is categorised as DCIS within a papilloma.^1^, ^3^ Mapping atypia with imaging is not feasible at present. Radiologically, intraductal papillomas are hypoechogenic lesions that are generally well‐circumscribed but can have irregular borders that may mimic carcinoma. Papillary lesions with atypia have reported upgrade rates as high as 47.8%,^1^ whereas papillary lesions without atypia have reported upgrade rates of 4%–7%.^7^, ^8^

Aside from the upgrade risk of the index lesion, the lifelong risk of developing breast cancer in either or both breasts should also guide management decisions.^1^ There is little evidence to suggest that RS/CSL or papillary lesions without atypia are premalignant lesions; both are associated with a two‐fold increased risk of breast cancer when compared to the general population.^8^, ^9^, ^10^ At present, it is recommended that patients with RS/CSL or papillary lesions without atypia on diagnostic biopsy have subsequent VAE followed by 3‐yearly mammography.^1^ However, there is some evidence that suggests that if these lesions are diagnosed using VAB, excision could be safely avoided.^11^

To address this, we reviewed all cases of RS/CSL and papillary lesions without atypia diagnosed and managed at King's College Hospital (KCH), either through the Southeast London Breast Screening Service or the symptomatic clinic, between January 2017 and June 2023, to determine the upgrade rates following both immediate excision and longer follow‐up.

## Methods

### Cohort

KCH pathology and breast cancer screening computer databases were searched to identify patients who received a needle biopsy diagnosis of ‘radial scar’, ‘complex sclerosing lesion’ or ‘papilloma’ between January 2017 and June 2023 (AL). Cases with atypia, as well as cases where the entire lesion (measuring <2 mm on microscopic examination) was removed on biopsy (resulting in a B2 classification as per current Royal College of Pathologists guidelines^2^, ^12^), were excluded from the study. Clinical outcome data were obtained from the electronic medical record and breast cancer screening database.

For each case, the following clinico‐pathological data were recorded: age of patient at diagnosis; indication for biopsy (screening or symptomatic); type of biopsy (CNB or VAB); size of the lesion on ultrasound and/or mammogram; biopsy diagnosis; B code; type of excision; and diagnosis on excision. At the time of histopathological diagnosis, all cases were reviewed routinely by a second specialist breast pathologist prior to discussion at the multidisciplinary team (MDT) meeting. The radiological images for those patients where the lesion size was not recorded at diagnosis were reviewed and documented during the study by two consultant breast radiologists (AB and JM). An ‘upgrade’ was defined as the presence of DCIS or invasive breast carcinoma (IBC) on excision at the biopsy site.

### Statistical Analysis

All statistical analysis was performed on GraphPad Prism V 10.4.1. Pairs of unmatched non‐parametric data were compared using a Mann–Whitney *U*‐test. A *P*‐value of ≤0.05 was considered significant.

## Results

**Figure 1:**
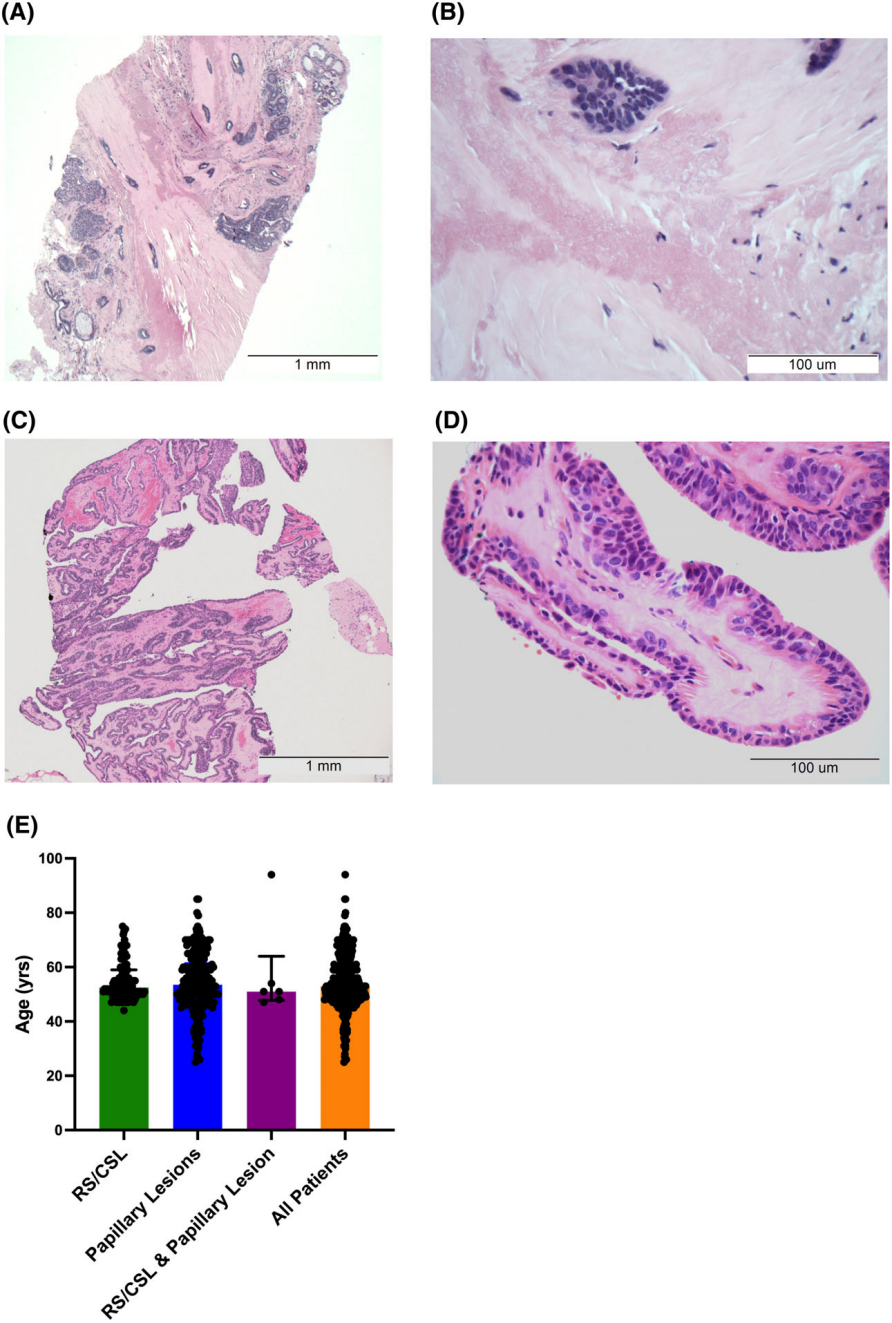
Histology of, and age at diagnosis for, RS/CSL and papillary lesions without atypia. A representative photomicrograph of an RS/CSL without atypia is shown in (**A**) (×40 magnification). The fibroelastotic stroma with entrapped glands is highlighted in (**B**) (×400 magnification). A representative photomicrograph of a papillary lesion without atypia is shown in (**C**) (×40 magnification). A single papilla comprising a central fibrovascular core, lined by a benign epithelial‐myoepithelial bilayer, is highlighted in (**D**) (×400 magnification). There was no significant difference in the median age (53 years) at diagnosis for either or both lesions (E).

Over the study period, 102 patients were diagnosed with RS/CSL without atypia (Figure 1A,B); 226 patients had papillary lesions without atypia (Figure 1C,D); and six patients were found to have both lesions on initial biopsy. The median age at diagnosis was similar for all lesions (Figure 1E; 53 years).

### RS/CSL Without Atypia

Of the 102 patients diagnosed with RS/CSL, 51% underwent a CNB, while 49% had a VAB. Most lesions were screen‐detected (85%); only 15% were symptomatic. Radiologically, 86 cases (84%) were biopsied since an RS/CSL was suspected. Of the remaining 16 cases (16%), 13 cases (13%) were sampled for indeterminate microcalcification, and in three cases (3%), a spiculate mass was targeted.

**Figure 2:**
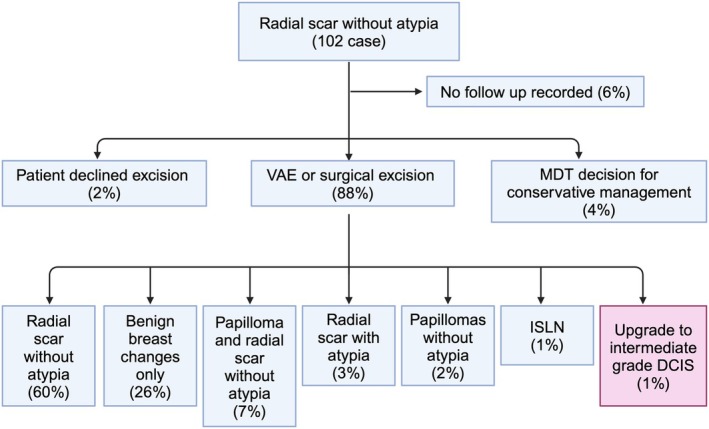
Cohort and outcomes of patients diagnosed with RS/CSL without atypia (n = 102).

After diagnosis (Figure 2), 90 patients (88%) went on to have excisions (85% VAE; 3% surgical); four patients (4%) opted for conservative management after MDT discussion; two patients (2%) declined further treatment; and six patients (6%) were lost to follow‐up. Of the 90 patients who underwent excisions, only one case was upgraded to intermediate‐grade DCIS (3 mm), i.e. an upgrade rate of 1%. There were no upgrades to invasive carcinoma. In addition, one patient had lobular neoplasia in situ (LCIS), and a further three patients (3%) were found to have RS/CSL with atypia not amounting to DCIS (i.e. either atypical ductal hyperplasia (ADH) or atypical intraductal epithelial proliferation (AIDEP)) in their excision specimens. For the latter three patients, annual mammography was recommended by the MDT, and there have been no upgrades to date.

**Figure 3:**
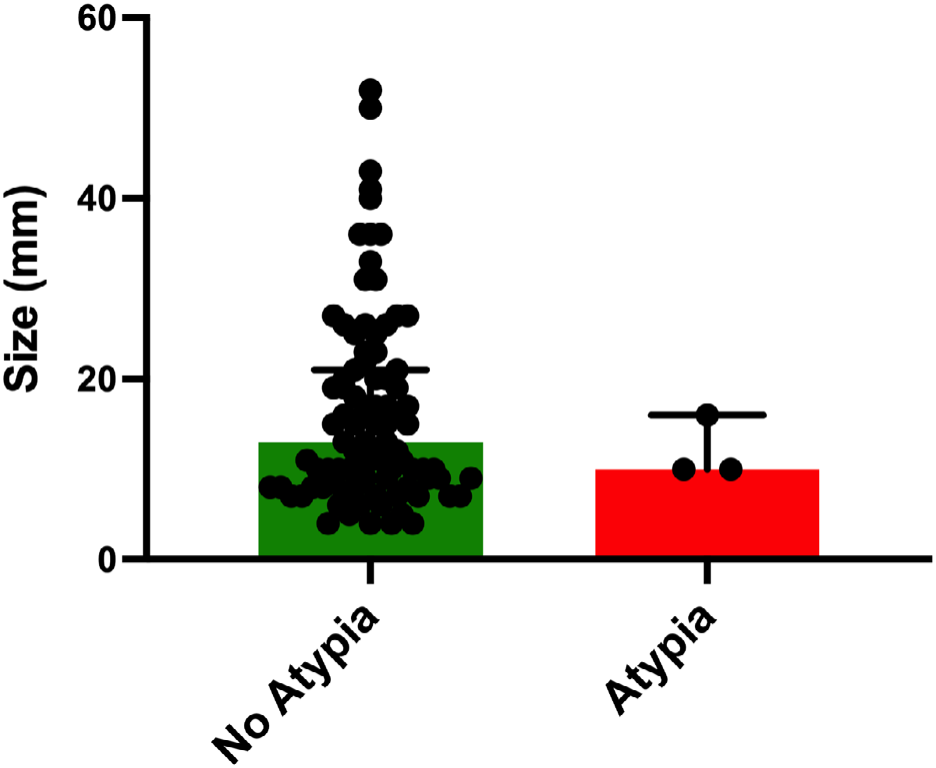
Radiological size comparison between RS/CSL without atypia and RS/CSL with atypia after excision. There was no significant difference in size between RS/CSL without atypia after excision (*n* = 92; median size = 13 mm) and RS/CSL with atypia after excision (*n* = 3; median size = 10 mm) in this cohort. Mann–Whitney *U*‐test (*P* ≤ 0.05 considered significant).

After radiological review of the diagnostic mammograms, size data were available for 97 patients (95%). This showed that there was no significant difference in size between RS/CSL without atypia (*n* = 92; median size = 13 mm) versus RS/CSL with atypia (*n* = 3; median size = 10 mm; Figure 3). The two cases where LCIS or DCIS was identified on excision were excluded from this analysis, since they were reclassified as malignant.

On follow‐up, one patient (1%) developed high‐grade DCIS in a different quadrant of the ipsilateral breast 3 years later. In addition, two patients (2%) developed invasive cancer in the same area of the ipsilateral breast, one within 3 years and the other within 5 years. All of these patients had their initial RS/CSL treated with VAE. Finally, one patient was found to have high‐grade DCIS with microinvasion in the contralateral breast. This patient had opted for conservative management.

### Papillary Lesions Without Atypia

Of the 226 patients diagnosed with papillary lesions without atypia, 94 (42%) were screen‐detected lesions and 132 (58%) were symptomatic. Of these, 196 (87%) of patients were diagnosed by CNB, as opposed to VAB (13%). Radiologically, 213 cases (94%) were biopsied since a papillary lesion was suspected. The remaining 13 cases (6%) were all sampled for indeterminate microcalcification.

**Figure 4:**
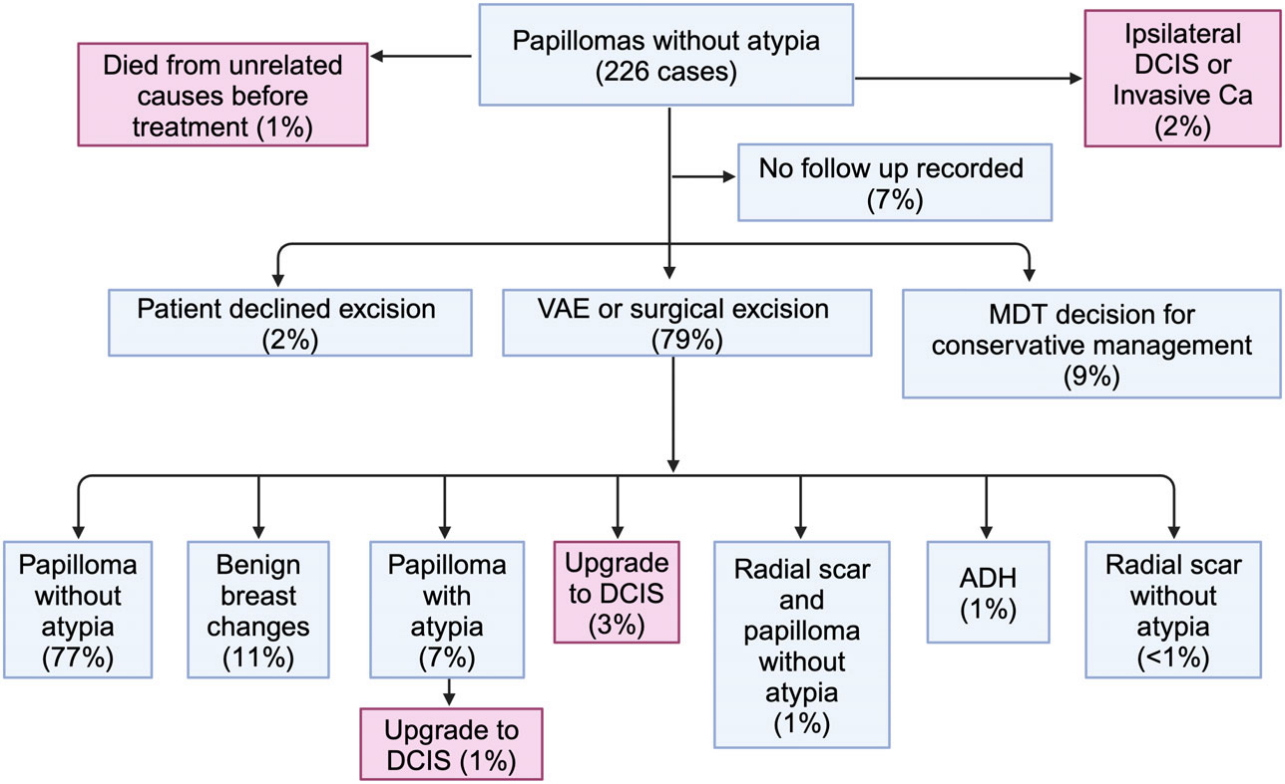
Cohort and outcomes of patients diagnosed with papillary lesions without atypia (*n* = 226).

After diagnosis (Figure 4), 15 patients (7%) were lost to follow‐up; four patients (2%) declined excisions; and five patients (2%) were found to have ipsilateral DCIS or invasive carcinoma in addition to their papillary lesions and were managed accordingly. Furthermore, 21 patients (9%) opted for conservative management (discussed below).

In total, 179 patients (79%) underwent an excision. Of these, 139 patients (78%) were treated by VAE, 36 patients (20%) underwent an open surgical biopsy and four patients (2%) had a microdochectomy. After excision, six patients were found to have DCIS, that is an upgrade rate of 3%. Two of these cases were screen‐detected and four were symptomatic. There were no upgrades to invasive carcinoma. Twelve patients (7%) had papillary lesions with atypia not amounting to DCIS (i.e. ADH or AIDEP) in their excisions. Most of these patients were followed up with annual mammography. However, one symptomatic patient had multiple papillomas in the same breast and went on to have surgery, where 3 mm of intermediate‐grade DCIS was identified in that breast. A second symptomatic patient went on to have a WLE that showed low‐ and intermediate‐grade DCIS; this patient also had a concurrent, contralateral invasive breast cancer. Thus, taking these two cases into consideration, the overall upgrade rate for papillary lesions without atypia was 4% (two screen‐detected cases (2%) and six symptomatic (4.5%)). Interestingly, one patient in this cohort developed ipsilateral, high‐grade DCIS 2 years later. One patient was lost to follow‐up. The remaining 10 patients are currently on 5‐year annual mammographic surveillance. Seven have had normal scans so far, but three have had further papillary lesions diagnosed in the same breast.

**Figure 5:**
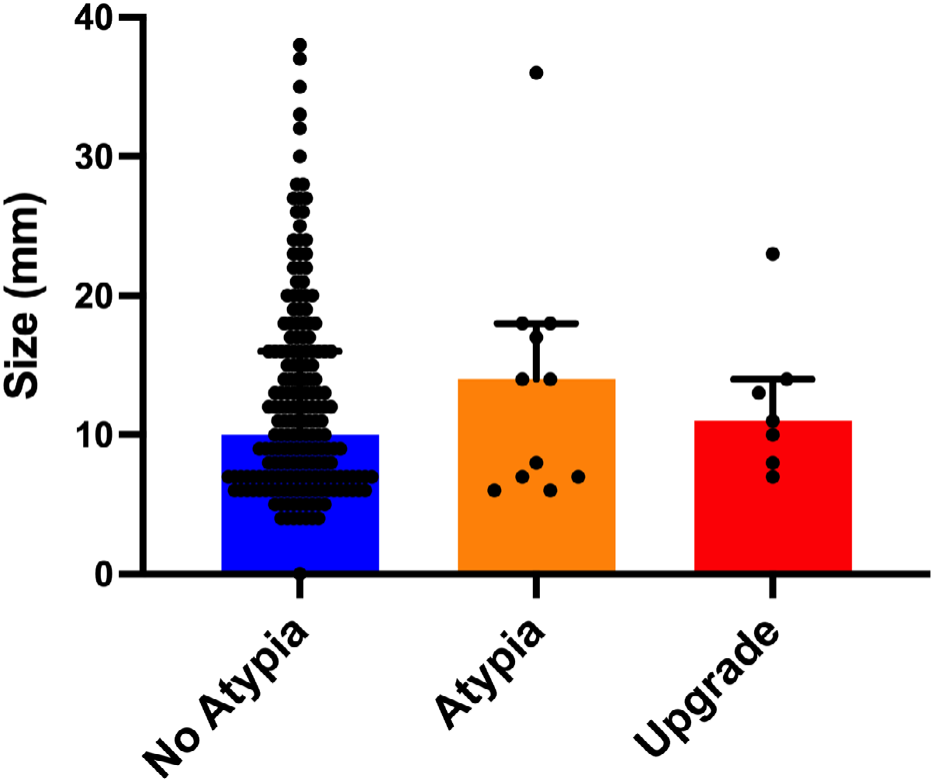
Radiological size comparison between papillary lesions without atypia and papillary lesions with atypia or an upgrade to DCIS after excision. There was no significant difference in size between papillary lesions without atypia (*n* = 190; median size = 10 mm) versus papillary lesions with atypia (*n* = 11; median size = 14 mm) and/or those lesions that were upgraded after excision (*n* = 7; median size = 11 mm). Mann‐Whitney *U*‐test (*P* ≤ 0.05 considered significant).

After radiological review of the diagnostic ultrasounds, size data were available for 202 patients (89%). Mammographic lesion size was available for a further six patients (3%). There was no significant difference in size between papillary lesions without atypia (*n* = 190; median size = 10 mm) versus papillary lesions with atypia (*n* = 11; median size = 14 mm) and/or those lesions that were upgraded after excision (*n* = 7; median size = 11 mm; Figure 5).

Of the 21 patients (9%) who were managed conservatively, 13 (62%) have had stable scans thus far. One patient's lesion increased in size – she ultimately opted for surgical excision, which confirmed a papilloma without atypia, i.e. no upgrade was found. One patient went on to develop invasive breast cancer in the contralateral breast; five patients were lost to follow‐up, and one patient died from an unrelated cause.

### RS/CSL and Papillary Lesion Without Atypia

Six patients had both an RS/CSL without atypia and a papillary lesion without atypia in their initial biopsy. Five of these patients underwent excision; no upgrades were detected.

## Discussion

While it is widely accepted that the upgrade risk of RS/CSL or papillary lesions with atypia warrants excision, how best to manage these lesions in the absence of atypia is less clear. This is complicated by the fact that the recording of associated atypia in B3 lesions within the NHS Breast Screening Programme is variable, thus confounding how national pathology audit data is analysed and interpreted.^13^ Since data entry has been cited as the major cause for this variation,^13^ we decided to audit these lesions in‐house over a 7‐year period.

The upgrade risk in RS/CSL without atypia in our cohort was very low (1%). Considering that there was an even split between the number of patients diagnosed on CNB versus those diagnosed on VAB, it seems unlikely that this low upgrade rate is due to larger amounts of tissue being sampled upfront. Interestingly, most of these lesions were screen‐detected (85%), and it seems intuitive that this could have influenced the upgrade rate. However, there was no statistically significant difference in size observed between RS/CSL with or without atypia in this series. Could it be, therefore, that in the absence of atypia, RS/CSL are really a benign entity that can be managed conservatively? The fact that a further 4% of patients subsequently developed malignant lesions in the ipsilateral breast (and predominantly in the same area of the breast) during follow‐up argues against this conclusion somewhat. Reassuringly, though, all of these malignant lesions were detected on mammographic surveillance. This suggests that RS/CSL without atypia can be routinely observed, rather than excised.^14^ More data from other screening centres is needed, however, before this change can be considered and/or implemented.

On the other hand, papillary lesions without atypia appear to be more complex, and unfortunately, once again, size does not appear to be predictive of atypia in this cohort. It is, however, noteworthy that the risk of upgrade to DCIS in the symptomatic cohort (4.5%) was twice that of screening patients (2%), suggesting that screening does decrease risk in these lesions even in the absence of atypia. Interestingly, most patients (87%) in our cohort were diagnosed on CNB rather than VAB. This does raise the question of whether obtaining more biopsy tissue would increase diagnostic accuracy in assessing atypia, especially since these lesions are known to show intralesional heterogeneity.^15^ We are considering trialling at KCH, and if this holds true, this could help with risk stratifying these patients and lowering the upgrade rate (4% in this cohort). The confounding effect of intralesional heterogeneity on fine‐tuning management was also evident in the fact that in those patients where atypia was diagnosed on excision, one patient went on to develop ipsilateral malignancy within 2 years, and three patients had further papillary lesions diagnosed in the same breast. In addition, while many patients who opted for conservative management in this group had stable disease on follow‐up (62%), one patient did go on to develop contralateral invasive breast cancer, whilst another HAD surgery since the lesion became larger. Therefore, the risk of malignancy both ‘now’ and ‘later’ in this cohort was high enough to preclude a change in current management guidelines, i.e. these lesions should continue to be excised until more data becomes available.

## Conclusion

Over a 7‐year period, we found that RS/CSL without atypia carry a low upgrade rate (1%). This suggests that these lesions could be managed conservatively, with mammographic surveillance only rather than VAE. Further data from other UK centres are needed, however, before this change in practice can be considered. On the other hand, papillary lesions without atypia carry an upgrade rate of 4%. This suggests that VAE is required in the management of these heterogeneous lesions going forward.

## Author contributions

KN conceived the audit; AL collected the data; AB and JM reviewed the radiological images and measured lesion size; AL and KN analysed the data; AL, AB, JM and KN wrote and edited the manuscript.

## Funding information

Not applicable.

## Conflict of interests

The authors declare that they have no competing interests.

## Data Availability

All data generated or analysed during this study is included in this published article (and its supplementary information files).
